# Inferring cancer dependencies on metabolic genes from large-scale genetic screens

**DOI:** 10.1186/s12915-019-0654-4

**Published:** 2019-04-30

**Authors:** Shoval Lagziel, Won Dong Lee, Tomer Shlomi

**Affiliations:** 1 Faculty of Computer Science, Technion, Haifa, Israel; 2 Faculty of Biology, Technion, Haifa, Israel; 3 Lokey Center for Life Science and Engineering, Technion, Haifa, Israel

**Keywords:** Cancer metabolism, Gene-silencing screens, RNAi, CRISPR, Tumor microenvironment, Tissue culture medium, Metabolic networks

## Abstract

**Background:**

Cancer cells reprogram their metabolism to survive and propagate. Thus, targeting metabolic rewiring in tumors is a promising therapeutic strategy. Genome-wide RNAi and CRISPR screens are powerful tools for identifying genes essential for cancer cell proliferation and survival. Integrating loss-of-function genetic screens with genomics and transcriptomics datasets reveals molecular mechanisms that underlie cancer cell dependence on specific genes; though explaining cell line-specific essentiality of metabolic genes was recently shown to be especially challenging.

**Results:**

We find that variability in tissue culture medium between cell lines in a genetic screen is a major confounding factor affecting cell line-specific essentiality of metabolic genes—while, quite surprisingly, not being previously accounted for. Additionally, we find that altered expression level of a metabolic gene in a certain cell line is less indicative of its essentiality than for other genes. However, cell line-specific essentiality of metabolic genes is significantly correlated with changes in the expression of neighboring enzymes in the metabolic network. Utilizing a machine learning method that accounts for tissue culture media and functional association between neighboring enzymes, we generated predictive models for cancer cell line-specific dependence on 162 metabolic genes (representing a ~ 2.2-fold increase compared to previous studies). The generated predictive models reveal numerous novel associations between molecular features and cell line-specific dependency on metabolic genes. Specifically, we demonstrate how cancer cell dependence on one-carbon metabolic enzymes is explained based on cancer lineage, oncogenic mutations, and RNA expression of neighboring enzymes.

**Conclusions:**

Considering culture media as well as accounting for molecular features of functionally related metabolic enzymes in a metabolic network significantly improves our understanding of cancer cell line-specific dependence on metabolic genes. We expect our approach and predictive models of metabolic gene essentiality to be a useful tool for investigating metabolic abnormalities in cancer.

**Electronic supplementary material:**

The online version of this article (10.1186/s12915-019-0654-4) contains supplementary material, which is available to authorized users.

## Background

Metabolic reprogramming is an emerging hallmark of cancer [1]. Evidence for alterations in metabolic activity in malignant cells goes back almost a century ago to the discovery of the “Warburg effect” [2]. The recent resurgence of interest in the field of cancer metabolism involves numerous findings of metabolic alterations in a variety of pathways in cancer cells [3]. These metabolic adaptations are directly regulated by growth-promoting signaling pathways, in which somatic mutations drive tumorigenesis [4]. Multiple drugs targeting metabolic enzymes have been used in clinics for decades, and many promising agents are under clinical trials [5–7]. Major ongoing research is aimed to identify tumor-specific reliance on metabolic enzymes that are limiting for cancer progression and to exploit these liabilities to treat cancer [5].

Genome-scale RNAi and CRISPR-based gene silencing-screens are powerful tools to identify essential genes for cancer cell proliferation and survival [8–13]. Recent pan-cancer loss-of-function screens of hundreds of cancer cell lines have identified novel oncogenes involved in cell signaling pathways. Integration of measured gene essentiality profiles with genomic features (DNA mutations, copy number variations, and RNA expression) was shown to provide a mechanistic understanding and predictive models for tumor-specific gene essentiality [14–16].

Explaining cell line dependence specifically on metabolic enzyme-coding genes appears to be particularly challenging. For instance, in a recent large-scale RNAi screen by Tsherniak et al. [14], the number of generated predictive models for cell line-specific dependence on metabolic genes was significantly lower (by ~ 30%) than for non-metabolic genes (hypergeometric *p* value < 10^−4^). In the genetic screen by McDonald et al. [15], a similar difficulty to predict cell line-specific dependence on metabolic genes was shown. A notable example of cell line-specific dependency on a metabolic enzyme-coding gene that is explainable based on gene expression is that of TYMS (thymidylate synthase) [15], where TYMS was found to be essential in cell lines where its expression level is low (hence, cells may be limited for thymidylate needed for DNA biosynthesis) while the expression of TYMP (thymidine phosphorylase) is high (further depleting thymidylate pools).

Here, we aimed to generate predictive models for cell line-specific dependence on metabolic genes based on genomic features, providing insight into the underlying molecular mechanisms. Considering that cellular metabolism is highly dependent on the availability of metabolic nutrients in the environment [17–19], we further accounted for the variability in tissue culture medium used for different cell lines in the genetic screens—a confounding factor that appears to have a major effect on cell line-specific essentiality of metabolic genes, thought quite surprisingly, has not been previously accounted for. We show that considering culture media as well as accounting for the functional association between metabolic enzymes via a metabolic network model enables generating predictive models for cancer cell line-specific dependence on a ~ 2.2-fold higher number of metabolic genes than in previous studies.

## Results

### Metabolic gene essentiality depends on tissue culture medium

**Fig. 1:**
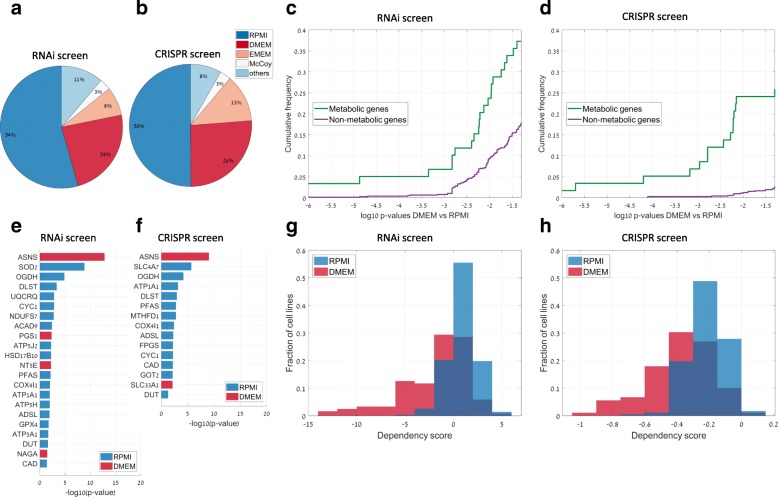
Tissue culture media significantly affect cancer cell dependence on metabolic enzymes. **a**, **b** The distribution of culture media types throughout cell lines used in large-scale RNAi genetic screen by Tsherniak et al. (**a**) and in a CRISPR-based screen by Meyers et al. (**b**). **c**, **d** Difference in RNAi (**c**)- and CRISPR (**d**)-based dependency scores of metabolic genes between cell lines grown in DMEM versus in RPMI (green) versus for other genes (purple), showing the cumulative distribution of FDR-corrected Wilcoxon *p* values. **e**, **f** Metabolic genes whose cell line-specific dependence is significantly correlated with the utilized tissue culture media in the RNAi (**e**) and CRISPR (**f**) screens. Blue and red represent genes that were found to be more essential in RPMI and DMEM, respectively. **g**, **h** The distribution of RNAi (**g**)- and CRISPR (**h**)-based dependency scores for ASNS (asparagine synthetase) throughout cell lines grown in DMEM versus in RPMI

Considering that metabolic activity is highly dependent on nutrient availability, we hypothesized that variability in culture media used for different cell lines in genetic screens may have a profound effect on cellular dependence on specific metabolic genes. To test this hypothesis, we utilized data from recent large-scale RNAi and CRISPR screens (covering 501 and 341 cell lines, respectively, as part of the Cancer Dependency Map) [14, 16]. Analyzing the reported information on tissue culture media in both screens showed that multiple media were used for different cell lines within each study (Fig. 1a, b). The two most commonly used media in both studies were RPMI and DMEM, in which > 50% and > 20% of the cell lines were cultured, respectively. RPMI and DMEM differ in terms of their composition of inorganic salts, amino acids, vitamins, and metabolic nutrients. For instance, RPMI includes several non-essential amino acids such as proline, aspartate, and asparagine which are absent from DMEM [20].

We compared the distribution of gene dependency scores of metabolic genes for cell lines grown in DMEM and RPMI (considering genes included in the metabolic network model Recon 2.2 [21]). We focused on metabolic genes whose dependency scores showed major variation throughout the cell lines, considering those whose dependency score in at least one cell line is lower by more than six standard deviations from the mean of each gene as in [14] (a low dependency score of a gene in a certain cell line reflects increased essentiality). We find that the RNAi-based dependency scores of 37% (22/59) of the analyzed metabolic genes are significantly associated with the utilized culture media (FDR-corrected Wilcoxon *p* value < 0.05) versus 18% of the non-metabolic genes (135/754, Fig. 1c). Similarly, we find that utilized culture media are significantly correlated with CRISPR-based dependency scores for a markedly higher percent of the metabolic genes (26%, 15/58 genes) versus other genes (3%, 19/716 genes; Fig. 1d). The metabolic gene that showed the strongest dependence on culture media in both RNAi and CRISPR screens was ASNS (asparagine synthase, Fig. 1e, f) and found to be significantly more essential in cells grown in DMEM (Fig. 1g, h; FDR-corrected Wilcoxon *p* value < 10^−12^ and 10^−9^ for the RNAi and CRISPR screens, respectively, comparing the distribution of dependency scores in cell lines grown in DMEM and RPMI). This is readily explainable by the lack of asparagine in DMEM, rendering ASNS essential for de novo asparagine biosynthesis under this culture media. Furthermore, ASNS was found to be significantly more important in cell lines grown in DMEM than those grown with other asparagine containing media, McCoy (FDR-corrected Wilcoxon *p* value < 10^−3^ for both RNAi and CRISPR screens), and Hams F12 (*p* value < 10^−6^ for both screens). 

Overall, we found a significantly stronger association between metabolic gene dependency scores and culture media versus that of non-metabolic genes and media (Wilcoxon *p* value < 10^−2^ for the RNAi screen and *p* value < 10^−4^ for the CRISPR screen, comparing the distribution of FDR-corrected Wilcoxon *p* values computed above for metabolic versus non-metabolic genes; Fig. 1b).

We next examined whether the identified correlation between metabolic gene dependency scores and culture media can be explained by variation in cell culture type (i.e., adherent versus suspension) or cancer lineage. Notably, DMEM was initially developed for culturing adherent cells [22] while RPMI was introduced for suspension cells [23]. Indeed, we found that ~ 90% and ~ 30% of the adherent and suspension cell lines from the genetic screens were grown in DMEM and RPMI, respectively. However, the correlation between metabolic gene dependency scores and media type remained highly significant when controlling for the cell culture type; for ~ 90% of the metabolic genes whose RNAi- or CRISPR-based dependency score correlate with media type, the correlation remains significant when controlling for cell culture type (FDR-corrected Spearman partial correlation *p* value < 0.05). Likewise, the correlation between gene dependency scores and media type remained significant for 64% of the cases when controlling for cancer lineage (see the “Methods” section; FDR-corrected *p* value < 0.05). Taken together, our results suggest that culture media is an important confounding factor affecting metabolic gene essentiality in RNAi- and CRISPR-based screens.

### Metabolic gene essentiality depends on the expression level of neighboring enzymes in the metabolic network

**Fig. 2:**
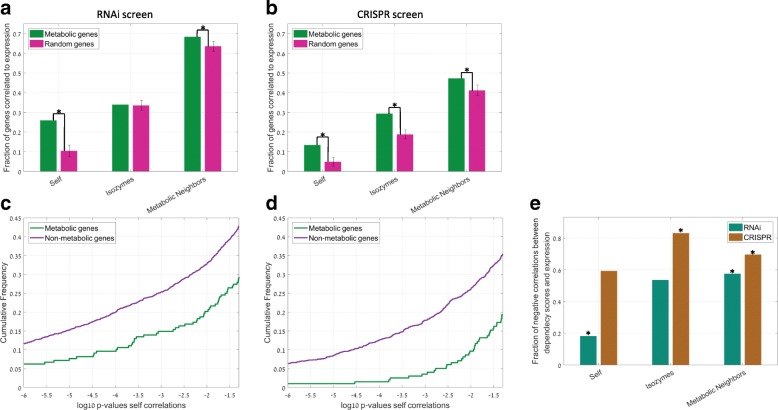
Cell line-specific dependence on metabolic enzymes is explainable by the expression level of neighboring enzymes in the metabolic network. **a**, **b** The fraction of metabolic genes whose cell line-specific RNAi (**a**) and CRISPR (**b**) gene dependency scores are significantly correlated with their expression, with the expression of an isozyme and with the expression of a neighboring enzyme in the metabolic network (shown in green) versus the fraction of metabolic genes with such significant correlations when randomly shuffling the expression measurements across genes (in pink). **c**, **d** The correlation between gene expression level and RNAi (**c**)- and CRISPR (**d**)-based dependency scores of metabolic (green) versus non-metabolic (purple) genes; showing the cumulative distribution of FDR-corrected Spearman *p* values. **e** The fraction of negative Spearman correlations (out of all significant correlations; positive or negative) between RNAi (green)- and CRISPR (brown)-based gene dependency scores and the expression level of the gene, that of an isozyme, or a neighboring enzyme. The asterisk marks a statistically significant high number of positive correlations (binomial *p* value < 0.05)

We explored whether cell line-specific dependence on metabolic genes is explainable based on altered RNA expression level. We find that RNAi- and CRISPR-based dependency scores of 26% and 14% of the metabolic genes, respectively, are significantly correlated with their expression level across cell lines (FDR-corrected Spearman correlation *p* value < 0.05; with the *r* value having an either positive or negative sign). This is significantly higher than expected by chance, when considering correlations between gene dependency scores and the expression of randomly chosen genes (*p* value < 0.05, Fig. 2a, b). However, the fraction of metabolic genes whose cell line-specific dependence is predictable by their expression level is significantly lower (by ~ 40%) than for non-metabolic genes (Wilcoxon *p* value < 10^−4^ for the RNAi screen and *p* value < 10^−7^ for the CRISPR screen; comparing the distribution of FDR-corrected Wilcoxon *p* values computed for metabolic versus non-metabolic genes; Fig. 2c, d). Interestingly, RNAi-based gene dependency scores and gene expressions are negatively correlated for a significantly small fraction of the metabolic genes (in ~ 20% of the cases; binomial *p* value < 10^−5^, assuming a probability of 0.5 for a positive correlation; Fig. 2e); that is, a negative correlation representing high essentiality in cell lines in which their expression level is high. With CRISPR, on the other hand, metabolic gene dependency scores were negatively correlated with the gene expression in ~ 60% of the cases. These results suggest that RNAi-based knockdown is especially effective in cell lines where the expression level of the target metabolic gene is already low, while CRISPR-based gene knockout affects cell viability regardless of the gene expression level.

Considering that metabolic flux typically depends on the collective activity of multiple interconnected enzymes (rather than the expression of a single enzyme), we hypothesized that cell line dependence on a specific metabolic gene might be explained by altered expression of neighboring enzymes in the metabolic network. To test this hypothesis, we computed the Spearman correlation between the RNAi- or CRISPR-based metabolic gene dependency scores and expression levels of isozymes and neighboring enzymes in the metabolic network (utilizing Recon 2.02) [21]. Specifically, we consider *neighboring enzymes* as those producing or consuming a shared metabolite, excluding those sharing highly prevalent currency metabolites such as energy or redox co-factors (see the “Methods” section). We find that the RNAi- and CRISPR-based dependency scores of a significantly high fraction of the metabolic genes are correlated with the expression level of at least one isozyme (for CRISPR screen) and with the expression of at least one neighboring enzyme (for both RNAi and CRISPR screens, *p* value < 0.05; Fig. 2a, b). Accordingly, we find higher Spearman correlations between gene dependency scores and the expression level of isozymes versus with other genes (*p* value < 10^−2^ for the RNAi screen and *p* value < 10^−26^ for the CRISPR screen) and with neighboring enzymes versus with other genes (*p* value < 10^−6^ for the RNAi screen and *p* value < 10^−26^ for the CRISPR screen; Wilcoxon test comparing the two distributions of absolute Spearman *r* values). A significantly high negative correlation between both RNAi- and CRISPR-based gene dependency scores and the expression levels of neighboring enzymes (binomial *p* value < 10^−5^) represent increased reliance on metabolic genes in cell lines having induced expression level of interconnected enzymes.

### Generating predictive models for cell line-specific dependence on metabolic genes accounting for culture medium and neighboring enzymes

Having found a significant correlation between metabolic gene essentiality and culture medium, and with the expression of functionally related enzymes in the metabolic network, we aimed to generate improved predictive models for cell line-specific dependence on metabolic genes. For each metabolic gene, we build a predictive model considering expression and copy number of functionally related metabolic genes (i.e., considering isozymes and neighboring enzymes in the metabolic network), somatic mutation of 125 known oncogenes/tumor suppressors [24], and culture media type. We utilized a bootstrap-aggregated random forest algorithm [25, 26] (similar to [14]; see the “Methods” section). The goodness of fit was evaluated based on the Pearson correlation between out-of-bag predicted and measured knockdown effects, estimating statistical significance via a random permutation test of dependency scores (see the “Methods” section).

**Fig. 3:**
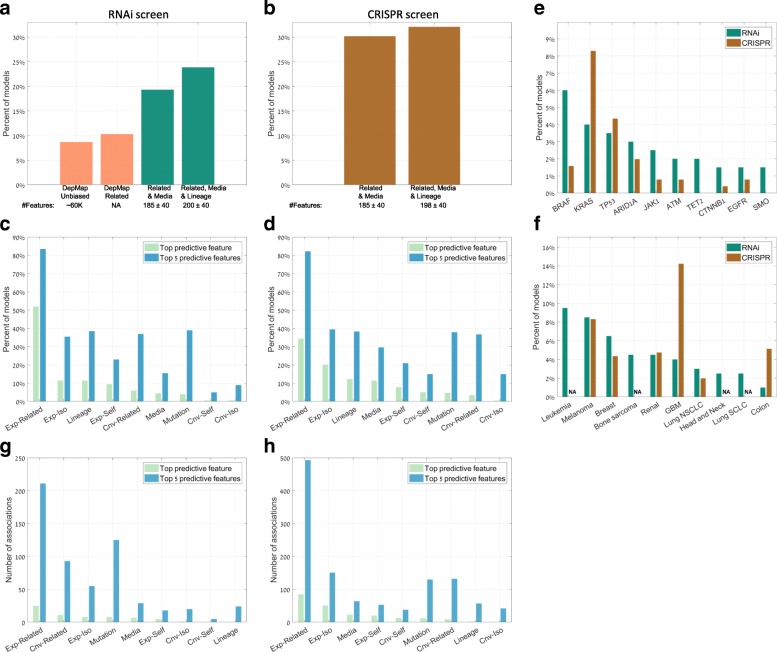
Predictive models for cell line-specific dependence on metabolic genes combining multiple molecular features. **a**, **b** The fraction of metabolic genes for which a significant predictive model of RNAi (**a**)- and CRISPR (**b**)-based gene dependency was generated by focusing on molecular features of neighboring enzymes and culture media and when also considering cancer lineage information (green for RNAi, brown for CRISPR). In comparison, the number of generated predictive models for RNAi-based gene dependency scores of metabolic genes derived by the Dependency Map project (based on molecular featured of all genes and using related genes) is shown in orange. **c**, **d** The prevalence of various types of features as the top (green) or within the top 5 (blue) features in the generated predictive models of RNAi (**c**)- and CRISPR (**d**)-based dependency scores of metabolic genes. “Exp” and “Cnv” are used as the abbreviations for gene expression and copy number variation, respectively. “Iso” and “Related” are used as abbreviations for an association of the target gene with its isozyme and related gene (i.e., neighboring enzyme), respectively. **e**, **f** The prevalence of specific oncogenic mutations (**e**) and cancer lineages (**f**) within the top 5 features in the generated predictive models for RNAi (green)- and CRISPR (brown)-based metabolic gene dependency scores. **g**, **h** The number of the top (green) or within the top 5 (blue) features in the generated predictive models that are not individually correlated with the RNAi (**g**)- and CRISPR (**h**)-based dependency scores

Our analysis resulted in significant predictive models for RNAi-based dependency scores for 19% of the analyzed metabolic genes (162 out of 838) (FDR-corrected *p* value < 0.05; Fig. 3a), with a mean Spearman correlation between predicted and measured dependency scores of ~ 0.37. This represents ~ 2.2-fold higher number of metabolic genes with a significant predictive model than those previously found via an unbiased approach as part of the Dependency Map project [14]. Notably, Tsherniak et al. repeated their analysis focusing on molecular features of functionally related genes (protein-protein interaction [27] or members of a protein complex [28]) to reduce the feature space and avoid overfitting. However, this increased the number of metabolic genes for which significant predictive models were generated by only 11% (while increasing the total number of genes with a predictive model by up to 19%). Hence, while focusing on molecular features of functionally related genes is clearly useful to increase the predictive power of gene dependence, the functional association of metabolic genes is better captured by our neighboring enzymes measure. Further considering that cellular metabolism is highly dependent on cancer lineage [29, 30], accounting for the information on cancer lineage markedly increased the number of significant predictive models for metabolic gene dependency to 24% of the metabolic genes (Fig. 3a, Additional file 1). Similarly, our analysis resulted in significant predictive models for CRISPR-based dependency scores for 32% of the metabolic genes (Fig. 3b, Additional files 2, 3, 4 and 5).

Next, we explored which type of molecular feature contributes the most to explaining metabolic gene dependency by performing permutation-based feature importance test on the derived predictive models from RNAi and CRISPR data (see the “Methods” section). We find that the expression level of neighboring enzymes is the top predictive feature of gene dependency in both RNAi (~ 50%) and CRISPR (34%) screens (Fig. 3c, d). Surprisingly, the expression level of the gene itself is the top predictive feature of RNAi- and CRISPR-based dependency scores in only ~ 10% and 8% of the metabolic genes, respectively, emphasizing the importance of analyzing the expression of interconnected enzymes in the metabolic network. Media type is the top predictive feature for another ~ 5% and ~ 11% of the metabolic genes with the RNAi and CRISPR screens, respectively. In comparison, oncogenic mutations were found to be the top predictive feature of RNAi- and CRISPR-based gene dependency for less than 5% of the metabolic genes (though oncogenic mutations have important predictive value when combined with other molecular features; among the top 5 predictive features of up to 40% of the metabolic genes).

Among the known oncogenic mutations, BRAF and KRAS mutations are the most frequently observed alterations within the top 5 predictive features of the RNAi-based (6%) and CRISPR-based (8%) metabolic gene dependency (Fig. 3e), followed by TP53 (~ 4%) for both screens. This is in agreement with major metabolic reprogramming known to be regulated by oncogenic mutations in these genes [31–34]. Among cancer lineages, leukemia is the most frequent cancer type within the top 5 predictive features of the RNAi screen (10%), followed by melanoma in both screens (8%, Fig. 3f).

Integrating multiple molecular and cancer lineage features within a predictive model for gene dependency reveals numerous important predictive features that cannot otherwise be inferred when analyzing each feature individually (Fig. 3g, h). For example, for 18 metabolic genes, we find that their gene expression level is an important predictive feature of RNAi-based cell line dependence (within the top 5 predictive features), while no significant correlation is found between their expression and dependency score across all cell lines. We find that the top predictive feature of RNAi-based cell line dependency for DAD1 (involved in *N*-glycan synthesis) is its expression level, while no significant correlation is found between DAD1 expression level and its dependency score across all cell lines (Spearman *r* = 0.03, *p* value = 0.57). Considering that the mutation status of NOTCH1 is also within the top 5 predictive features of DAD1, we find a significant correlation between DAD1 expression and its dependency score when focusing on NOTCH1-mutated cell lines (Spearman *r* = − 0.35, *p* value < 0.05). This is consistent with the known important role of glycosylation in Notch signaling [35], suggesting that in cancer cell driven by NOTCH1 mutation, high DAD1 expression level indicates high dependence on its function.

**Fig. 4:**
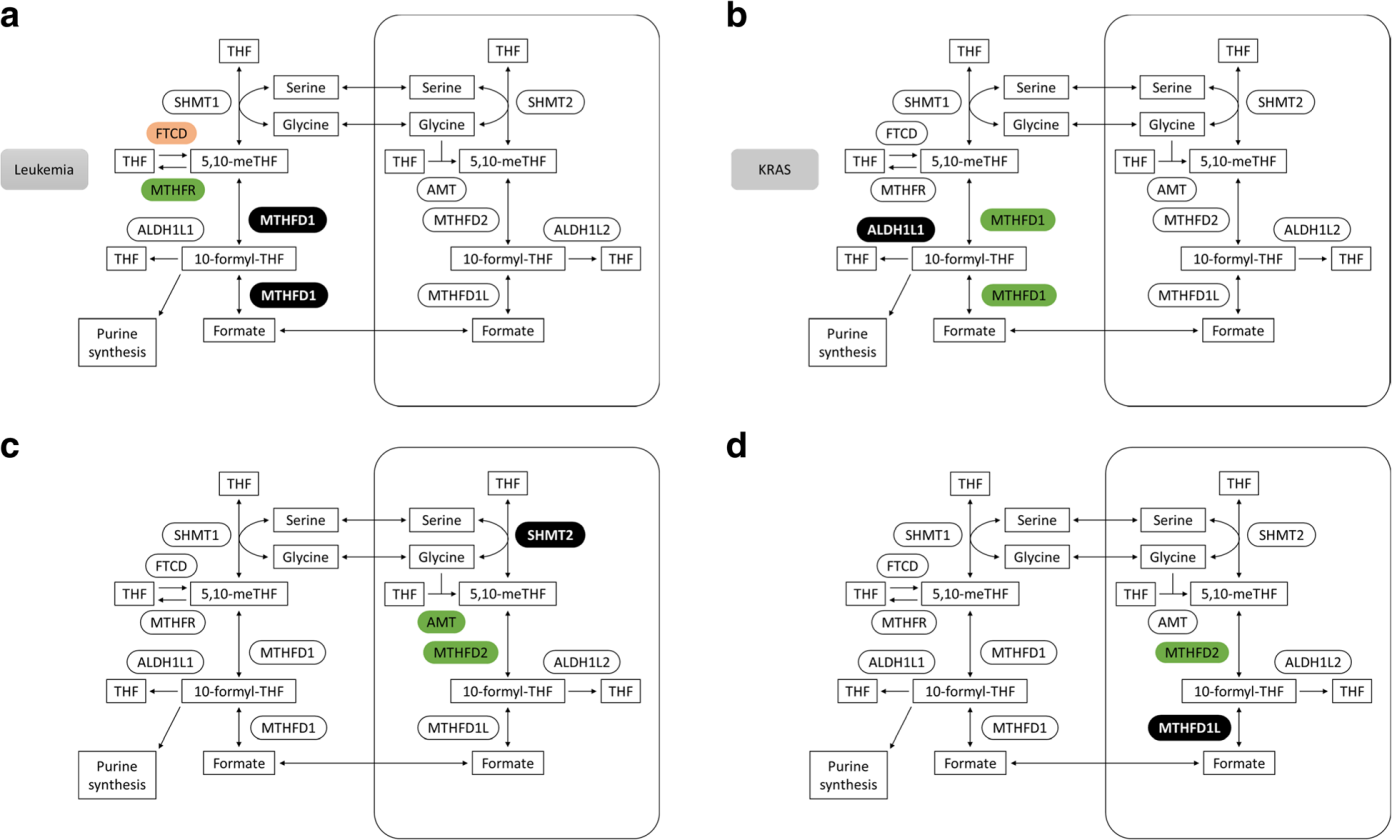
Predictive models for cell line-specific dependence on genes in one-carbon metabolism. Predictive models for RNAi-based dependency scores of MTHFD1 (**a**), ALDH1L1 (**b**), SHMT2 (**c**), and MTHFD1L (**d**). Significant predictive features of induced cell line-specific dependency are shown for each model (increased expression or copy number in green; decreased expression or copy number in orange)

The generated predictive models enable raising hypotheses regarding specific metabolic mechanisms that underlie cell line-specific dependence on metabolic genes. For example, we present predictive models generated for RNAi-based dependency on genes involved in one-carbon metabolism. This metabolic system has long been a target for chemotherapy and has recently been shown to involve substantial metabolic rewiring that can be targeted for therapeutic purposes [36–41]. We find an increased dependency of leukemia cell lines on MTHFD1 (cytosolic methylene-tetrahydrofolate dehydrogenase) where the expression of MTHFR (methylene-THF reductase) that consumes 5,10-methylene-THF is high, and the expression of FTCD (formimidoyltransferase cyclodeaminase) that produces 5,10-methylene-THF as part of histidine catabolism is low (Fig. 4a). This suggests that maintaining the high activity of MTHFD1 is required to overcome a potential depletion of its substrate 5,10-methylene-THF in these conditions. Increased dependence on ALDH1L1 (cytosolic formyltetrahydrofolate dehydrogenase) is found in KRAS-mutated cell lines where MTHFD1 copy number is high, potentially to facilitate the regeneration of THF from 10-formyl-THF (Fig. 4b). Increased dependency on SHMT2 (mitochondrial serine-hydroxymethyl-transferase) is found in cell lines with increased expression of AMT (a subunit of the glycine-cleavage system; localized in mitochondria) and of MTHFD2 (mitochondrial methylene-tetrahydrofolate dehydrogenase, Fig. 4c). This might occur in the face of a high demand rate for mitochondrial one-carbon units, requiring both serine and glycine catabolic activities. In cells where the expression of MTHFD2 is high, increased dependency on MTHFD1L might be needed to support mitochondrial formate production from 10-formyl-THF, facilitating the shuttling of one-carbon units to cytosol to support purine and pyrimidine biosynthesis [42, 43] (Fig. 4d).

## Discussion

A genome-scale genetic screen is a powerful approach for unbiased discovery of vulnerabilities in cancer cells that can be targeted for therapeutic purposes. Since the development of RNAi- and CRISPR-based gene-silencing techniques, both approaches have been systematically applied for screening the gene knockdown and knockout effects in numerous cell lines. To identify genes whose perturbation would selectively affect cancer cells, gene-silencing profiles are typically integrated with tumor-specific mutations and alterations in gene expression to identify a specific molecular marker indicative of cancer cell dependence on a target gene. This is typically referred to as synthetic lethality or synthetic dosage lethality, where a perturbation of a single gene is viable but the perturbation of both genes is lethal to cells [44]. The identification of such synthetic lethal gene pairs provides a patient stratification strategy based on the mutation or expression status of one gene where the drug targeting of its partner gene enables selective eradication of cancer cells while minimizing toxic side effects. Previous studies suggest going beyond the search for synthetic lethal pairs, generating predictive models of gene essentiality in cancer cells based on multiple molecular features [14, 15]. However, as shown here, the ability to generate such predictive models for metabolic genes was significantly limited compared to other non-metabolic genes.

Analyzing genetic silencing screen data from recent RNAi and CRISPR screens, we found a confounding effect of culture media on cell line-specific response to the silencing of especially metabolic genes. Our finding is consistent with recent studies, demonstrating the major effect of the tumor microenvironment in determining the cancer cellular metabolic phenotypes [17, 45–47]. In fact, several recent attempts have been made to develop culture media which would more closely mimic nutrient availability and concentration as in human plasma [20]. Our results, showing that the specific choice of media affects cell line dependence on metabolic genes, call for future genetic screens to maintain a similar culture media for different cell lines, optimally matching the true physiological conditions. Predictive models for cell line dependence on specific genes are not only important for potential usage as a patient stratification tool, but also useful for studying the molecular mechanisms that underlie cell line-specific metabolic activities. Hence, taking into account the culture media in our analysis, the generated predictive models are readily available for researchers interested in exploring cancer cell line dependence on specific metabolic genes. As shown, the integration of multiple molecular and cancer lineage features within a predictive model for gene dependency reveals numerous important predictive molecular features that cannot otherwise be inferred.

Another factor that complicates the finding of molecular features explaining cell line-specific gene dependence is the substantially high number of candidate predictive features: Specifically, the ability to identify synthetic lethality by analyzing the correlation between the dependency score of a certain gene and a large set of molecular feature suffers from multiple hypothesis testing, and the generation of predictive models via machine learning while considering a high number of features is complicated by overfitting. The incorporation of prior knowledge regarding feature relevance was previously shown highly useful for limiting the number of candidate predictive features and increasing statistical power [48]. Here, we show that a measure of functional relatedness of metabolic genes, defined based on connectivity in a metabolic network, is highly useful for generating predictive models for metabolic genes. Overall, utilizing this measure and also considering culture media composition, resulted in a 2.2-fold increase in the number of generated predictive models for metabolic genes compared to those derived as part of the Cancer Dependency Map [14].

Notably, while we were able to generate significant predictive models for cell line specific dependence on ~ 30% of the analyzed metabolic genes by integrating multiple molecular features, additional molecular characterization of the analyzed cell lines is expected to enable expanding this to other genes. Specifically, considering that metabolic activity is controlled beyond genomic and transcriptomic levels, additional high-throughput measurements of epigenetic modifications, proteomics, and metabolomics are expected to be highly useful in closing the gap.

## Conclusions

Culture media has a significant confounding effect on cell line-specific response to the silencing of metabolic genes, which should be taken into account when analyzing results from gene-silencing screens. Together with the media type, molecular features of functionally related enzymes in a metabolic network improve the prediction of cell line-specific dependence on metabolic genes. Overall, we expect our approach and predictive models of metabolic gene essentiality to be a useful tool for investigating metabolic abnormalities in cancer.

## Methods

### Pan-cancer gene essentiality screening data

The RNAi screen data analyzed in this study contains dependency scores for 17,098 genes over 501 cancer cell lines (released on July 2017) [14], while the CRISPR-Cas9 screen contains scores for 17,670 genes over 341 cancer cell lines (released on October 2017) [16].

### Statistical analysis of the correlation between gene dependency scores and culture media

To check whether cell culture type explains the correlation between media composition and dependency scores, we calculated for each gene the Spearman partial correlation between its RNAi dependency score across cell lines and a binary vector representing media composition in each cell line (DMEM or RPMI), controlling for binary vector representing cell culture type (suspension vs adherent). Derived *p* values were FDR-corrected via the method of Benjamini-Hochberg [49].

To test whether cancer cell lineage explains the correlation between media composition and dependency scores, we computed a similar Spearman partial correlation for each gene while controlling for the effect of an indicator variable representing each cancer lineage (having a value of one for cell lines of belonging to a specific lineage). We consider a correlation between gene dependency scores and media composition as not explainable based on cancer lineage information in case the above partial correlation remains significant when controlling for each cancer lineage (i.e., having an FDR-corrected *p* value < 0.05).

### Identifying neighboring enzymes in the human metabolic network

We consider a total of 1612 metabolic genes included in the genome-scale human metabolic network model Recon 2.02 [21] (omitting EGFR and PIK due to their central role in signaling pathways). The metabolic neighbors of a given metabolic enzyme are those that consume or produce substrate or product metabolites of this enzyme. Currency metabolites that participate in more than 1% of the reactions in the network (central energy or redox factors, etc.) were not considered to define metabolic neighbors. For each gene, we considered no more than 50 neighboring enzymes and those connected through reactants participating in the minimal number of metabolic reactions in the network.

### Pairwise correlations between gene dependency scores and expression of functionally related genes

We calculated the Spearman correlations between dependency scores of metabolic genes (focusing on those whose dependency score in at least one cell line is lower by more than four standard deviations from the mean of each gene) and the expression of 19,541 protein-coding genes derived from the CCLE [50]. Resulting *p* values were FDR-corrected using the method of Benjamini-Hochberg [49]. A similar analysis was performed using gene expression of isozymes or enzyme neighbors in the metabolic network. The dependency scores of a gene were considered to be explained by the expression levels of isozymes/metabolic neighbor in case a significant correlation (FDR-corrected *p* value < 0.05) is found for at least a single isozyme/metabolic neighbor. In order to check the statistical significance of the fraction of metabolic genes having a significant correlation, we performed a random permutation test repeating the analysis above 1000 times while randomly shuffling the set of neighbors/isozymes for each enzyme.

### Generation of predictive models of RNAi- and CRISPR-based gene dependency scores

We focus on metabolic genes from Recon 2.02 for which RNAi- or CRISPR-based dependency scores were available, and whose dependency scores showed considerable deviation throughout the cell lines (whose dependency score in at least one cell line is lower by more than two standard deviations from the mean of each gene). Molecular features, including RNA levels, copy number variation, and mutations in 125 oncogenes or tumor suppressors (non-synonymous point mutations, frameshift and splice mutations) [24], were derived from the CCLEs [50] and from CBioPortal [51, 52]. Information on media type and primary disease was derived from the screens data [14, 16]. Only cancer lineages having at least 10 cell lines in the screen were considered. To find predictive models, we use random forest learning [25, 26], generating an ensemble of 100 decision trees using MATLAB, with a min leaf size of 5. This method accommodates both continuous and categorical features, capturing nonlinear relationships and correcting for potential overfitting [53]. We perform out-of-bag prediction and record the Pearson correlation as the goodness of fit. We assessed the significance of the goodness of fit for each model by comparing its Pearson *p* value with a distribution of *p* values computed with randomly shuffled data (making 25K repetitions of selecting a gene by random, permuting its dependency scores, generating a predictive model, and computing the goodness of fit). To estimate the importance of the features used to learn the model, we calculated the out of bag permutated variable importance. To derive robust predictors, we selected the features with a positive feature importance for each model and regenerate that model by focusing on these features only (as in [14]). Extending the above analysis to include also copy number variation of oncogenes/tumor suppressors did not result in a higher number of significant predictive models for either RNAi or CRISPR screens.

## Additional files

Additional file 1: RNAi models. Excel table containing statistically significant predictive models found using the RNAi screen data. (XLSX 32 kb)

Additional file 2: CRISPR models. Excel table containing statistically significant predictive models found using the CRISPR screen. (XLSX 39 kb)

Additional file 3 **Figure S1.** *P* value distributions calculated as part of the generation of predictive models for metabolic gene dependency scores using expression/copy number variation of related metabolic genes, genomic mutations, media information, and cancer lineage for RNAi (a, c, e) and CRISPR (b, d, f) screens. (a, b) Distributions of *p* values obtained using randomly shuffled data: Selecting a gene by random, randomly permuting its dependency scores throughout the cell lines, generating a predictive model of its shuffled dependency scores throughout cell lines, and computing the goodness of fit between the generated model and shuffled dependency scores (25K repetitions). (c, d) Distributions of Pearson *p* values assessing the goodness of fit between dependency scores and model predictions. (e, f) Distributions of empirical *p* values after FDR correction: For each predictive model, an empirical *p* value is computed based on the fraction of Pearson *p* values obtained with random data (shown in panels c and d) that are equal or lower to the *p* value computed with the original data (and correcting for multiple hypothesis testing using the method of Benjamini-Hochberg). The black lines in subfigures c and d denote the threshold of significance (based on the distribution of *p* values generated with shuffled data; shown in panels a and b), and the lines in subfigures e and f denote a threshold of 0.05 on the empirical *p* values. (PNG 485 kb)

Additional file 4: **Figure S2.** Percent of statistically significant predictive models identified in our analysis using expression/copy number variation of related metabolic genes, genomic mutations, and media information (green); the percent of statistically significant predictive models using the same set of features though without media information (orange); and the percent of significant predictive models when randomly shuffling the set of related metabolic genes (i.e., for a given gene having N-related genes, N genes were randomly selected), repeating the analysis 100 times (purple). The latter was significantly lower than the percent of genes with a significant predictive model when considering all features (green) and without media information (orange; *p* value < 0.05, marked with an asterisk). (PNG 196 kb)

Additional file 5: **Figure S3.** (a, b) The fraction of metabolic genes (whose dependency score in at least one cell line is lower by more than six standard deviations from the mean of each gene) for which a significant predictive model of RNAi (a)- and CRISPR (b)-based gene dependency was generated by focusing on molecular features of neighboring enzymes and culture media and when also considering cancer lineage information (green for RNAi, brown for CRISPR). In comparison, the fraction of predictive models for RNAi-based gene dependency scores derived by the Dependency Map project (based on molecular featured of all genes and using functionally related genes) is shown in orange. (PNG 155 kb)
