# Supplementary figures and images for: Inferring cancer dependencies on metabolic genes from large-scale genetic screens
Source: BMC Biol. 2019 Apr 30;17:37. doi: 10.1186/s12915-019-0654-4 (PMC6489231; doi:10.1186/s12915-019-0654-4)

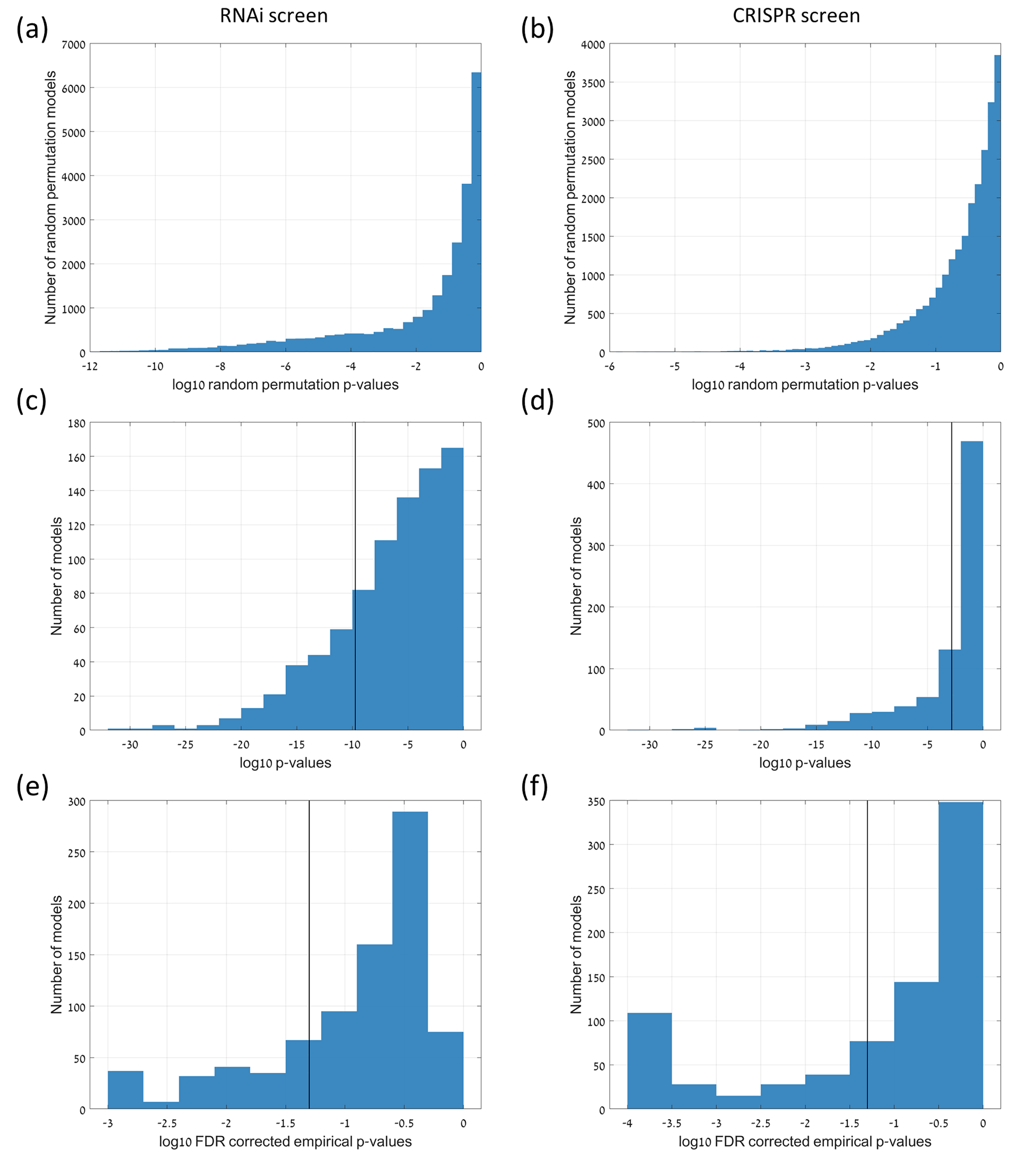

Supplement: Supplementary file 3 — Figure S1. P value distributions calculated as part of the generation of predictive models for metabolic gene dependency scores using expression/copy number variation of related metabolic genes, genomic mutations, media information, and cancer lineage for RNAi (a, c, e) and CRISPR (b, d, f) screens. (a, b) Distributions of p values obtained using randomly shuffled data: Selecting a gene by random, randomly permuting its dependency scores throughout the cell lines, generating a predictive model of its shuffled dependency scores throughout cell lines, and computing the goodness of fit between the generated model and shuffled dependency scores (25K repetitions). (c, d) Distributions of Pearson p values assessing the goodness of fit between dependency scores and model predictions. (e, f) Distributions of empirical p values after FDR correction: For each predictive model, an empirical p value is computed based on the fraction of Pearson p values obtained with random data (shown in panels c and d) that are equal or lower to the p value computed with the original data (and correcting for multiple hypothesis testing using the method of Benjamini-Hochberg). The black lines in subfigures c and d denote the threshold of significance (based on the distribution of p values generated with shuffled data; shown in panels a and b), and the lines in subfigures e and f denote a threshold of 0.05 on the empirical p values. (PNG 485 kb) [file 12915_2019_654_MOESM3_ESM.png]

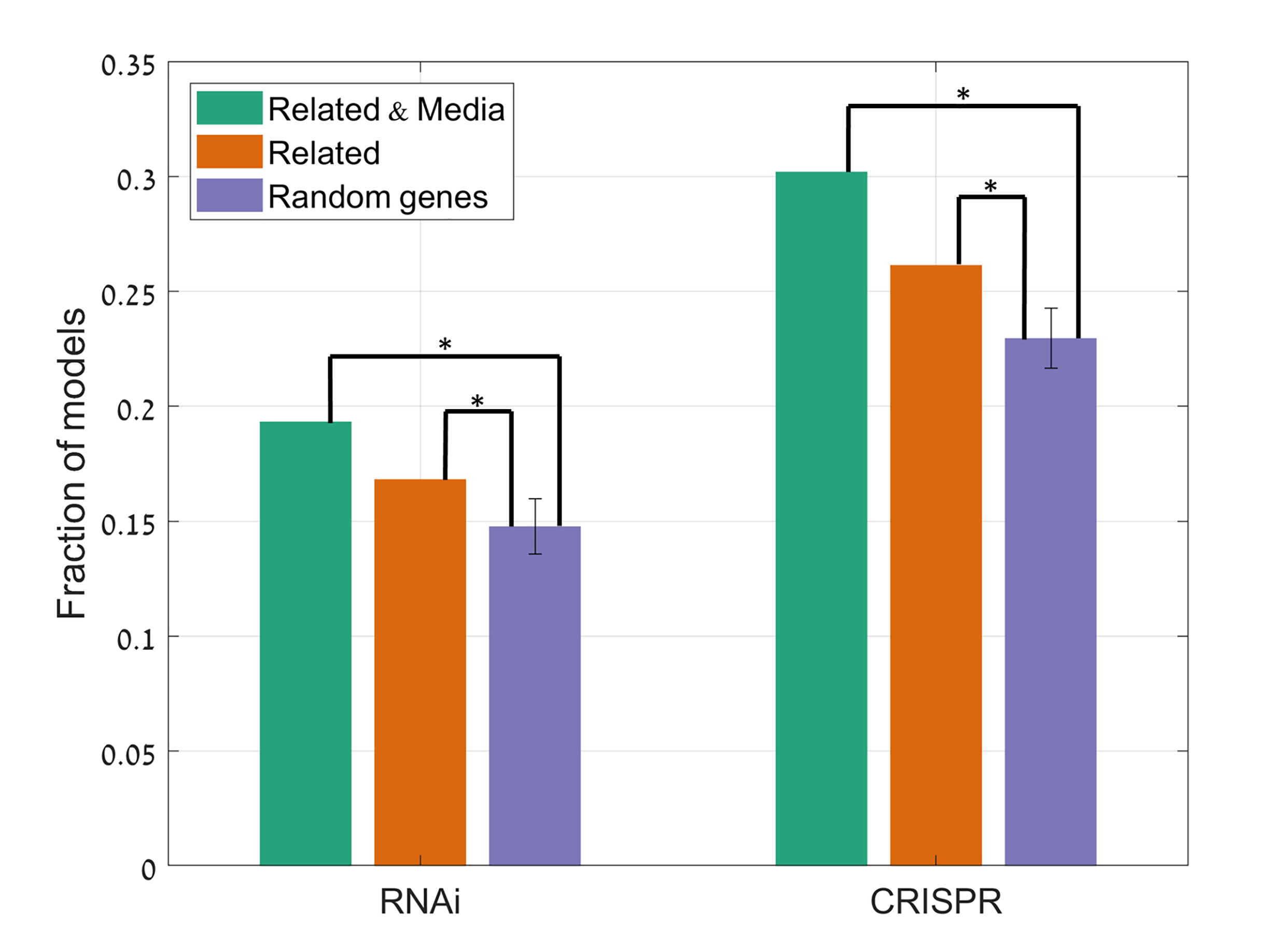

Supplement: Supplementary file 4 — Figure S2. Percent of statistically significant predictive models identified in our analysis using expression/copy number variation of related metabolic genes, genomic mutations, and media information (green); the percent of statistically significant predictive models using the same set of features though without media information (orange); and the percent of significant predictive models when randomly shuffling the set of related metabolic genes (i.e., for a given gene having N-related genes, N genes were randomly selected), repeating the analysis 100 times (purple). The latter was significantly lower than the percent of genes with a significant predictive model when considering all features (green) and without media information (orange; p value < 0.05, marked with an asterisk). (PNG 196 kb) [file 12915_2019_654_MOESM4_ESM.png]

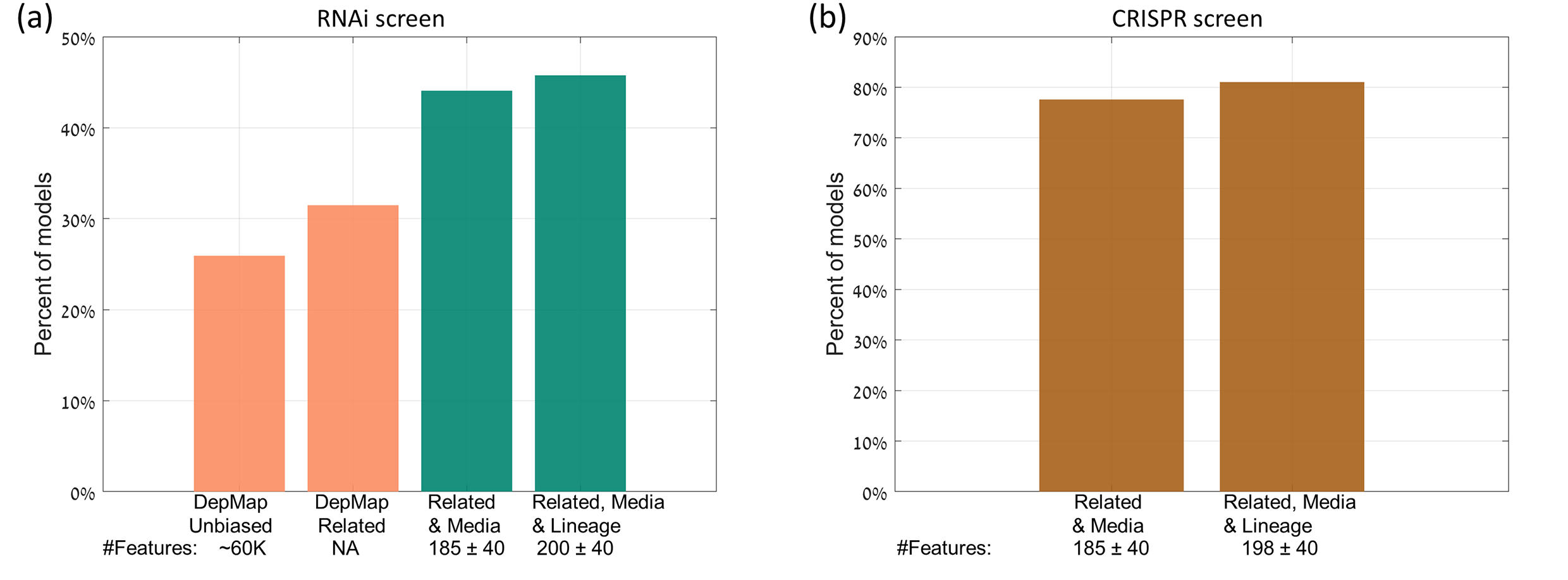

Supplement: Supplementary file 5 — Figure S3. (a, b) The fraction of metabolic genes (whose dependency score in at least one cell line is lower by more than six standard deviations from the mean of each gene) for which a significant predictive model of RNAi (a)- and CRISPR (b)-based gene dependency was generated by focusing on molecular features of neighboring enzymes and culture media and when also considering cancer lineage information (green for RNAi, brown for CRISPR). In comparison, the fraction of predictive models for RNAi-based gene dependency scores derived by the Dependency Map project (based on molecular featured of all genes and using functionally related genes) is shown in orange. (PNG 155 kb) [file 12915_2019_654_MOESM5_ESM.png]
